# COVID-19: A Curious Abettor in the Occurrence of Stevens-Johnson Syndrome

**DOI:** 10.7759/cureus.23562

**Published:** 2022-03-28

**Authors:** Dheera Grover, Meher Singha, Raj Parikh

**Affiliations:** 1 Department of Internal Medicine, University of Connecticut, Hartford, USA; 2 Department of Internal Medicne, University of Connecticut, Hartford, USA; 3 Department of Pulmonary and Critical Care Medicine, Hartford Hospital, Hartford, USA

**Keywords:** drug rash, rash, sjs, tamsulosin, toxic epidermal necrolysis (ten), covid 19

## Abstract

Stevens-Johnson Syndrome (SJS) and Toxic Epidermal Necrolysis (TEN) are immune-mediated life-threatening skin diseases. The condition is known to be caused by various infections, drugs (mainly antibiotics), or can be idiopathic. Amidst the novel coronavirus 2019 (COVID-19) pandemic, there is an increasing number of SJS/TEN cases being reported. Viral infections are known to have decreased the threshold of drug reactions by inducing a pro-inflammatory state in the body. We report a case of TEN secondary to tamsulosin use in the setting of COVID-19 infection. There is only one documented case of tamsulosin-induced SJS, and no documented case of TEN secondary to tamsulosin use. Our patient was a 26-year-old male who presented to the hospital after a recent history of COVID-19 infection with a diffuse maculo-vesicular rash with bullae, involving the mucosa. The patient had recent use of tamsulosin on the day of presentation and there were bullae and erythematous rashes present in the oral mucosa as well as significant conjunctival erythema with pain on ocular movement on physical examination. His rash progressively worsened, involving greater than 30% of his body. A biopsy was done that showed full-thickness necrosis indicative of toxic epidermal necrolysis (TEN). We hypothesize that in our patient COVID-19 infections lowered the threshold for the development of SJS/TEN.

## Introduction

Stevens-Johnson Syndrome (SJS) and Toxic Epidermal Necrolysis (TEN) are immune-mediated skin diseases characterized by widespread sloughing of the skin at the dermal-epidermal junction induced by drugs, infection, malignancy, or can be idiopathic [1]. Generally, TEN is a life-threatening severe cutaneous drug reaction, characterized by extensive epidermal detachment and mucositis, whose mortality depends on the SCORTEN scale [2]. These reactions are rare, with the rate of TEN being one to two cases per million [3,4]. Due to its potentially fatal nature, SJS/TEN is considered a medical emergency. Drugs are assumed to cause SJS/TEN, however, infections due to *Mycoplasma pneumoniae*, Herpes simplex virus have been known to cause it, and there are rare reports of coronavirus disease 2019 (COVID-19) causing SJS [5-7]. Allopurinol, trimethoprim-sulfamethoxazole, and other sulfonamide antibiotics, aminopenicillins, cephalosporins, phenobarbital, phenytoin, and carbamazepine are some common medications known to cause this reaction. Though SJS secondary to tamsulosin has been reported in the past, no literature is present on tamsulosin causing TEN [8]. We report a case of TEN in the setting of COVID-19 infection after the use of tamsulosin. Previous studies have shown a decreased threshold for the development of these reactions in inflammatory states such as infections [9]. There have been reports of a decreased threshold for the development of SJS/TEN in COVID-19 pneumonia as well.

## Case presentation

A 26-year-old male with no significant past medical history presented to the hospital with a recent COVID-19 infection followed by a diffuse maculo-vesicular rash with bullae involving the mucosa for two days. Twenty days prior to admission, the patient experienced symptoms of fever, headache, sore throat, dry cough, and fatigue and went to a drive-in COVID-19 polymerase chain reaction (PCR) testing site and was found to be positive for SARS-CoV-2 pneumonia. The next day, he presented to an outside hospital emergency department for a recurrent sore throat and was given a prescription for mouthwash and hydrocodone-acetaminophen. No antibiotics were given at this time. The patient also saw a urologist on the same day who ordered a prescription for tamsulosin for a new diagnosis of chronic urge incontinence that he had since childhood. He had never used tamsulosin in the past. One day after starting the new medications, the patient developed a progressively worsening rash involving the whole body including his chest, arms, palms, and soles (greater than 30%). The rash appeared to involve the mucosa and had multiple vesicles present. The patient was started on dexamethasone, remdesivir, and given one dose of intravenous immunoglobulin (IVIG) for concerns of vasculitis secondary to COVID-19 infection. The review of symptoms was positive for change in appetite, chills, diaphoresis, fatigue, fever, congestion, drooling, facial swelling, mouth sores, sore throat, and trouble swallowing. On presentation to our hospital, he was febrile to 103 degrees Fahrenheit, all other vitals were normal. There was significant conjunctival erythema without ocular motion pain and the patient’s vision was intact. 

**Figure 1:**
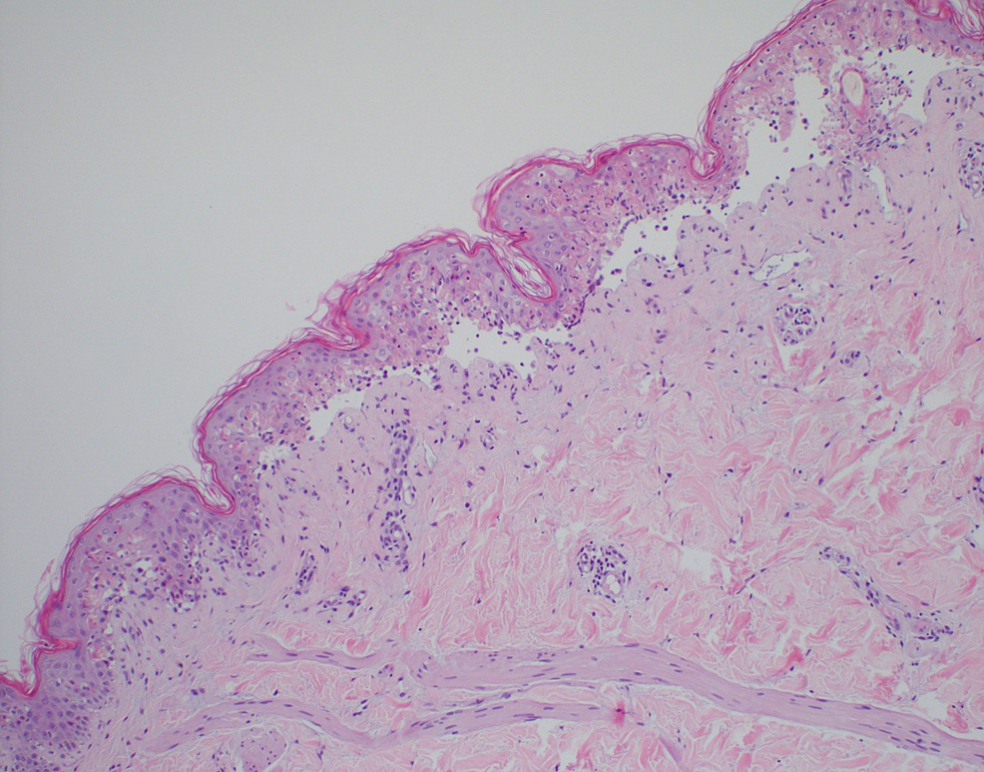
Micrographs showing epidermal necrosis with the lifting of epidermis on low power, findings compatible with TEN.

**Figure 2:**
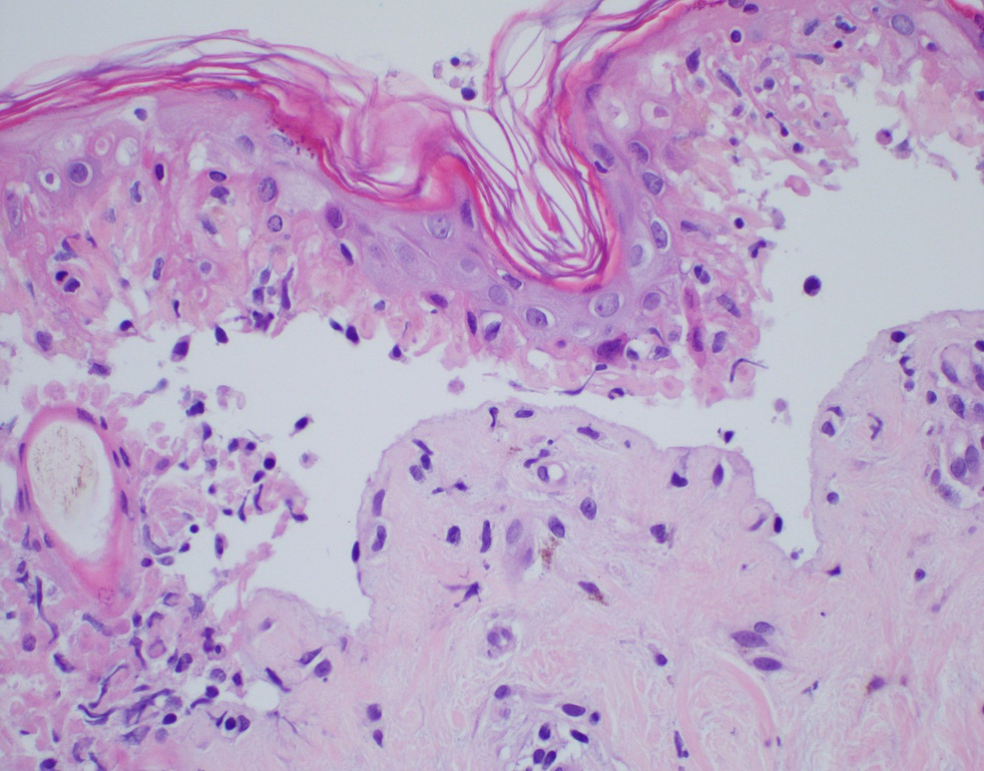
Micrographs showing epidermal necrosis with the lifting of epidermis on high power, findings compatible with TEN.

The patient was started on patient-controlled fentanyl to alleviate his pain in the emergency department. His laboratory values showed a white blood cell count of 3 x 1000/µL (normal range 4.0 - 10.0 x 1000/µL), neutrophil count 1.9 x 1000/µL (normal range 1.0 - 11.0 x 1000/µL) that decreased to 1.8 x 1000/µL during the hospital course, normal electrolytes, and renal function at the patient’s baseline with a creatinine of 0.6 mg/dL (normal range 0.40 - 1.30 mg/dL). He completed a course of IVIG and steroids for three days. He was also started on vancomycin and aztreonam. Infectious diseases, ophthalmology, and dermatology were consulted. A biopsy was done that confirmed the diagnosis of full-thickness necrosis indicative of toxic epidermal necrolysis (Figures 1, 2).

**Figure 3:**
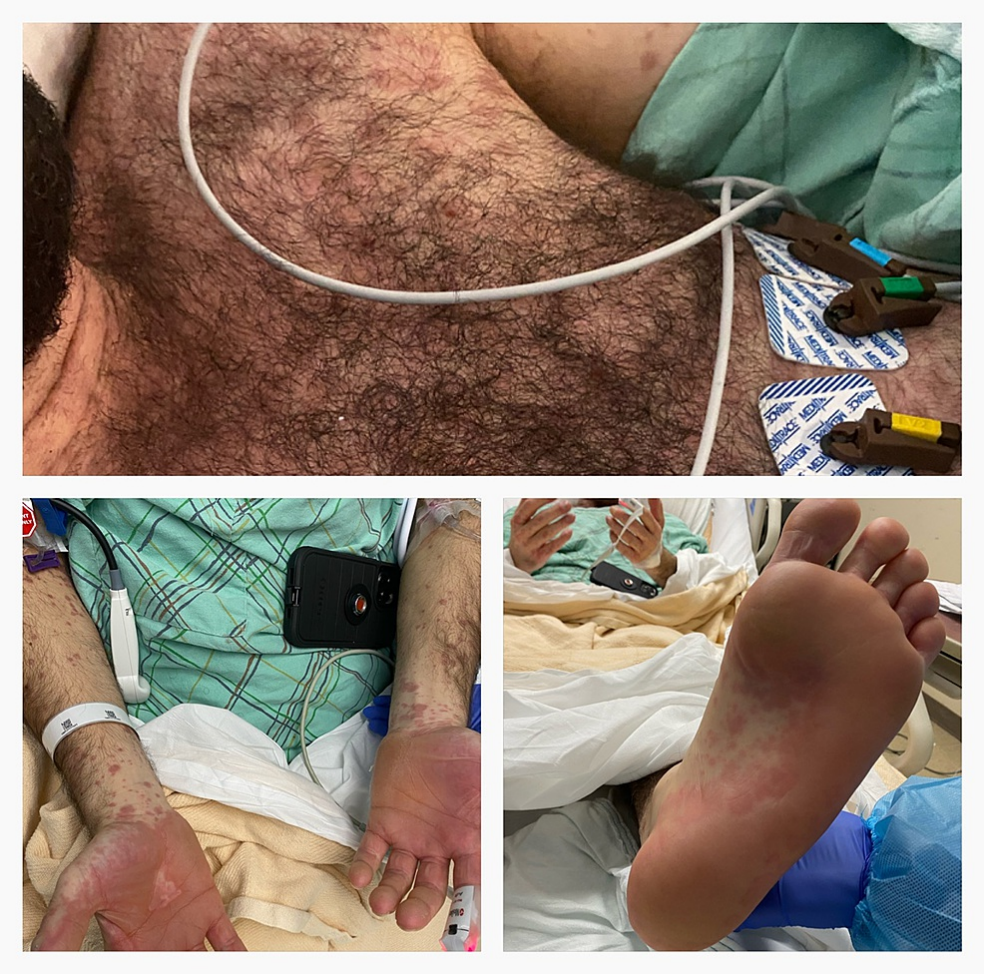
Pictorial representation of bullae on chest, hands, and soles.

During his hospital stay, the patient had worsening of his diffuse rash converging on the chest, large bullae covering the palmar aspect of his hands, plantar aspect of feet, bilateral ears, and blisters on his oral mucosa (Figure 3). Large bullae on the lateral right arm had a positive Nikolsky sign.

## Discussion

Undoubtedly, there is a lot to unravel about COVID-19 infection. The novel virus initially extracted from the lower respiratory tract and thought to only involve the respiratory tract has been seen to cause gastrointestinal, cardiovascular, cutaneous, neurological, and hematological manifestations as well. Cutaneous manifestations secondary to COVID-19 are rare and varied, including but not limited to erythematous rash, urticaria, chickenpox-like vesicles or pustules [10,11]. Based on our preexisting knowledge, the cutaneous manifestations in COVID-19 can be mainly due to underlying two mechanisms, as a response to viral particles or due to systemic inflammation leading to vasculitis or thrombosis [12]. Additionally, viral infections induce a pro-inflammatory state in the body and are known to predispose the patients to various cutaneous adverse reactions [9]. It becomes a clinical challenge to determine if the manifestations are primarily due to the COVID-19 infection or due to drugs being administered to the patient. We came across a patient who developed TEN secondary to tamsulosin and had a concomitant COVID-19 infection.

Tamsulosin has been reported to be a known culprit in causing SJS, but no cases have been reported of TEN secondary to the drug. So far, the cases reported of TEN in COVID-19 patients are secondary to the drugs reactions and not as a manifestation of the viral exanthem. To our knowledge, this is the first case reported of TEN secondary to tamsulosin. We speculate that the concomitant COVID-19 infection flared up the inflammatory skin reaction leading to clinical worsening. To determine the drug causality, the algorithm of drug causality for epidermal necrolysis (ALDEN) can be used [13]. The algorithm is a gold standard tool and helps in differentiating the culprit drug from innocent bystanders. It gives a score ranging from -12 to +10 to each drug, which corresponds to the probability of having caused the reaction, ranging from very unlikely to very probable. Unfortunately, due to the worsening clinical course, the ALDEN score was not calculated in our patient. 

According to a review of 42 patients conducted by Tran et al. in 2019, the mean age of presentation of SJS was 45.8 years compared to 54.9 years of TEN [8]. SJS was also noted to manifest in a shorter duration after commencement of drug as compared to TEN (12.6 vs 14.1 days). The exact pathology of SJS/TEN is unknown; however, it is thought to be due to T-cell mediated cytotoxic reaction to drug antigens in keratinocytes leading to blister formation [3]. The diagnosis is made by histopathologic and immunofluorescence examination on skin biopsy. The findings in the initial stages include scattered apoptotic keratinocytes in the basal layer of the epidermis, and perivascular, inflammatory infiltrate in papillary dermis primarily composing of T-lymphocytes [14]. The above findings are, however, not specific to SJS/TEN and can be found in other conditions including simple drug-induced exanthem. At later stages, there is frank development of subepidermal bullae with full-thickness epidermal necrosis. SJS/TEN are severe diseases with mortality rates ranging between 1-5% and 25-35% respectively [15]. Mortality of SJS/TEN can be calculated with the help of the SCORTEN score [16]. The score was initially developed to determine the in-patient hospital mortality in patients with TEN, however, is being used to calculate mortality in burn patients and several other cutaneous drug reactions as well [17]. The score weighs in 7 independent risk factors including age above 40 years, malignancy, heart rate greater than 120 beats per minute, initial percentage of epidermal detachment above 10%, serum urea above 10 mmol per liter, serum glucose above 14 mmol per liter, and bicarbonate below 20 mmol per liter. In our patient, the SCORTEN score at presentation was 1 indicating a 3.2 % mortality rate. 

Even after years of familiarity with the condition, the treatment of SJS/TEN still remains a clinical challenge. Based on the underlying pathogenesis, systemic corticosteroids and IVIG suppressing the excessive immune response remain the primary choice of treatment [18,19]. However, studies have shown that treating patients with immunosuppressive agents prolong the hospital stay, increase the risk of infections and increase mortality [20]. The above treatment acts as a double-edged sword in treatment patients with SJS/TEN with concomitant COVID-19 infection. 

## Conclusions

SJS/TEN are life threatening dermatological conditions which can be induced by infections, drugs, malignancy, or can be idiopathic. Amidst the pandemic, there have been rising number of cases of SJS/TEN in COVID-19 patients. It is important to carefully consider the various exposures which could be causing the condition and not attribute the causation to viral infection itself. The ALDEN algorithm should be followed to recognize the culprit drug to prevent further exposure. Further studies are required to better understand the cutaneous manifestations in the novel virus.
